# Important Conference of Non-Panel Practitioners

**Published:** 1913-12-06

**Authors:** 


### IMPORTANT CONFERENCE OF NON-PANEL PRACTITIONERS.

### The National Medical Union Formed.

### FIFTY NON-PANEL ASSOCIATIONS FEDERATED.

A Conference of medical men representing non-panel Associations from all parts of the country was held at the Waldorf Hotel, London, on Saturday, November 29, 1913.

Ninety-five delegates attended, representing some 50 Associations comprising over 1,000 members.

It was explained at the Conference by some of the speakers that non-panel Associations were being formed continuously in various places throughout the United Kingdom, but there had not been sufficient time or opportunity to prepare a complete list so as to include every such society. One result of the present Conference would be to afford every non-panel practitioner throughout the United Kingdom an opportunity to send in his name and join the National Medical Union. It was affirmed by more than one speaker that there must be at least ten thousand non-panel practitioners. It was agreed that the temporary offices of the Union should be at the Waldorf Hotel, Strand, London. All communications should be sent to the Honorary Secretaries at that address, until further notice.

Dr. J. S. Prowse, Manchester, as Secretary of the National Medical Union, which called the Conference together, welcomed the delegates.

On the motion of Dr. R. Carswell, Mr. William Coates, C.B., D.L., Chairman of the National Medical Union, took the chair.

On the motion of Dr. Wheeler Hart, Dr. R. Carswell and Dr. J. S. Prowse were appointed Secretaries to the Conference.

Dr. Prowse having read the notice convening the meeting,

### THE CHAIRMAN'S ADDRESS.

The Chairman, in opening the proceedings, referred to the circumstances under which the National Insurance Act came into force. He detailed the history of the fight made by the British Medical Association and sketched the circumstances attending the inception and development of the National Medical Union. It was only now, he said, they were beginning to realise that there was still something that could be done. There was at present no rallying ground over which non-panel practitioners could shake hands and become strong in this matter. They could see clearly that the Act was not only derogatory to the profession, but, as worked at the present time, was actually an absolute menace to the national health. The National Medical Union therefore felt that as there were 10,000 men in the country who were not serving on the various panels, this non-panel element of the profession might become really strong, and it was for that reason they had met on the present occasion. It should be remembered that they had not done with this Insurance Act. The present Government was not going to last for ever, and if another Government came in to-morrow non-panel practitioners would be able to do little if they were split up into small organisations, whereas if they were united as one solid body they could do a very great deal indeed. The British Medical Association was not a proper fighting force now for non-panel members. The British Medical Association could not serve two masters. These were the principles of those who had assembled at that Conference, but others might have other principles, so they had met to see if they could not bind together and help one another. It would be their proud privilege to combine to restore some of the old noble traditions under which their profession had worked, and as a learned profession to prove a source of strength to the national health.

### THE NATIONAL MEDICAL UNION FORMED.

Mr. G. A. Wright, F.R.C.S., moved the first resolution as follows:—

**"That this meeting of delegates from non-panel Medical Associations agrees to form an organisation with central offices in London by the federation of existing non-panel bodies."**

He said he took it that the object of the Conference was to consider what were the best methods of protecting the rights and interests of those members of the profession who did not desire to carry on their practice under the present conditions of the National Insurance Acts. Among the letters was one which proposed for their consideration the federation of the non-panel societies of which the names had been read out. It could not be expected that all these societies would have identical views as to what was desirable, nor would they all agree in regard to the means of carrying out their views, but there were certain resolutions before the Conference, and it was much to be desired that, if possible, unanimity should prevail with regard to them. For such a fusion as was proposed in the resolution he now moved it was hoped to attain an increasing power, together with simplicity in working; they aimed at bringing into one body all medical practitioners who did not wish to serve under the existing Acts. The great number of small local Associations could do little as they were, but they could do much through combination with a Central Council. If they were asked why they held out against the majority who were working the Acts, and why such an organisation as that now proposed was necessary, they might refer to the old discussions and resolutions of 1911-12 before the great collapse, and they might point to the constantly increasing number of Associations of non-panel men as a proof of the existence of serious evils in the present system. They might say now that the objections to the law as it stood fell into two classes—those which were concerned with the general principles of the Acts, and those concerned with medical benefits alone. The new organisation proposed, he believed, to deal only with the latter. Everyone, he supposed, from the post-office contributor to the author of the Acts, admitted that it was full of faults, and they had grounds for hoping that the next Government would drastically amend the law. The need for a powerful combination of non-panel organisations was shown by the attitude of the British Medical Association. This of itself would fully justify the formation of such a society as that now proposed for the protection of the interests of practitioners not working under the Acts. Such protection was undoubtedly needed against the effects of the Acts in depriving doctors of their practice on no other grounds than their refusal to join the panel. This refusal certainly did not justify a penalty of loss of livelihood, and an attempt to force men on to the panel by pressure either oblique or direct was grossly tyrannical. The loss of practice was not the only evil effect. The system itself led to a lamentable amount of ill-feeling between panel and non-panel practitioners. Again, it could not be denied that the system had actually and admittedly produced a quality of work in some cases worse than that of the old club practices and dispensaries, which it had superseded, and such a result seemed inevitable under existing methods. The question of provision for the care of "dependents" was one with which men not on the panel were deeply concerned, but he would not enter into that further than to point out how important it was that those who preferred not to work under the Acts should have a strong organisation at their backs to protect their interests. There were other reasons for the formation of such an organisation, but he would only point out that it was important that such a body should press for the formation of a Medical Committee, to which the draft of a new or amended Insurance Act should in due course be referred by the Government. An Insurance Act in some form was no doubt desirable, and no doubt would be a permanent institution, but the general approval of the medical profession was essential to its successful working, and if, while 15,000 or more approved, there were 10,000 who disapproved, all was not well. Their presence there was a proof that there was still a widespread and strong feeling in the profession against the provisions of the existing Acts, and there they were fortified by the knowledge that they shared those views with a large part of the community. They must recognise that if they were to give effect to their views, the immediately important business was the concentration of their forces, and he begged therefore to move the resolution.

Dr. H. B. Greene seconded the resolution.

In all this fight, he said, they had arrived now at the gravest and most critical point in its history. They were there at the wish of sporadic and scattered non-panel Associations which were in active existence in the country; how many these numbered he himself had little idea of until ten days ago. They were now federating at the wish of a number of non-panel Associations, who had come together like crystalline growths, forming bigger and bigger groups, but all were permeated by the common policy—non-panel. Dr. Greene referred with indignation to the treatment of the profession by politicians, and deprecated dependence on political views. It was known, he said, that the Act was favoured by the group of capitalists on either side of the House of Commons. The power of money would be brought forward to insure another thirty-one millions of women and children in order to bring another thirty-one millions of premiums into the coffers of a certain organised group of capitalists. The effect of this control of money was seen also in the Press. With one or two honourable exceptions the Press had not advocated the cause of the non-panel men as they had a right to expect. He hoped that they would be able to take back from that Conference the news that it was now known that these non-panel Societies were in existence, and that they were become a power to be reckoned with. If that power was to be effective it must be kept independent of any influence which was in any way under one or other political denomination. That was the reason that he held that the British Medical Association was not the proper body to organise the profession now; he held that it never was the proper body. He therefore advocated a Federation in which they would be loyal to each other, and which would prove above all that they were a profession.

The resolution was unanimously adopted.

### CONDITIONS OF MEMBERSHIP.

Dr. F. Porter proposed the second resolution:—

**"That the principal condition of membership of the new Association shall be the refusal of service, in any capacity, under the existing National Insurance Acts 1911 and 1913."**

Why, he asked, were they non-panel men? Because they refused to accept service under the Insurance Act. Why were local medical committees and Insurance Committees constituted? They were constituted to further the working of the Insurance Act. He could not understand the position of non-panel men who joined these committees. He had heard it said that they were to further the non-panel cause, but he had never heard of any assistance they had ever given in that direction. He should like to hear, therefore, why they were serving on these committees. He had heard it said also that they were on these committees for the purpose of serving good panel men; in doing that were they also serving non-panel men? Their first duty was to non-panel men, and for the non-panel cause. The medical men assembled at that Conference were now about to build a new edifice. Were they going to lay the foundations with a weak stone here and there, so that in a few years the edifice might totter to the ground? He hoped they would take a lesson from Germany; there was a precedent there which should warn them to guard against the German weakness. The position of the German profession to-day was absolutely chaotic, because they compromised. They were going on compromising; let English medical men take care that they did not drift in the same way; let them build their edifice strong now, and have no compromise. It would be better to begin with a small membership and gradually build up. In that way they would be strong. He repeated, no compromise or they were lost.

Dr. R. Carswell, in seconding the resolution, said the point of the resolution lay in the words "in any capacity." That was an uncompromising and absolute position to take up. The meeting was there to organise and to classify; to form and delineate a new species of medical organisation whose name should not be a byword. He referred to the rule in biology that in the separation and delineation of species all experience taught that it was better to draw the line ever too narrow than too wide, so as to escape the dangers and difficulties due to the inclusion of incompatible elements.

### THE CASE OF MEMBERS SERVING ON INSURANCE COMMITTEES.

Mr. E. B. Turner, Non-Panel Medical League, moved as an amendment to leave out all words after "shall be," and to substitute "that no member shall be on the panel or have a partner or assistant who is on the panel, or shall be a referee or accept any paid office under the National Insurance Acts 1911 and 1913."

He believed that if the resolution was carried as it stood the Conference would be making a mistake. All over the kingdom there were strong non-panel men who had taken office on Insurance Committees and Statutory Medical Committees who were doing what they could for the profession. This was not the time now that they were making a start to rule these men out of their membership. On the London Insurance Committee he himself was doing work which was a great deal more use to the profession than he would do by being a member of the Non-panel Practitioners' Association. The time was coming—the year after next—when in the first place what was described as the Chancellor's bribe would run out, and the whole question of the money would have to be rearranged, as it was only budgeted for up to that time. At the same time would come the revaluation of the Approved Societies. At the present moment there was hardly one keeping within the actuarial limits of the Act, and when the revaluation came he believed it would be found that they would have to make a levy on their members; then the *débâcle* would begin. The cry would be to cut down the doctors' remuneration, and unless the non-panel organisation was strong it would not be able to meet the situation. They wanted to rope in everybody, and this was not the time to begin by ruling out some of the men who were willing to do good work for them, while they also thought they could do good work for the profession generally by remaining on the Insurance Committees. On the London Insurance Committee they had succeeded in getting them to allow a large amount of contracting out. (A delegate: "That is not being done.") These men treating contracted-out patients were doing work actually under the Act, and if the Conference was to be logical under the wording of the original resolution, it would have to rule out these as members of the new Federation. If they did so they would be ruling out a large number of men who were heart and soul with them.

Dr. Montgomery Smith seconded the amendment. He said he would be in agreement with the resolution as printed on the agenda if he could see that it was possible to carry it out. If they could say that they had every non-panel man in their Federation they could dictate terms to anybody; but in changing their generalship he recommended the Conference to go a little slowly, and, above all, to show a united front. On these Insurance Committees they had some of their best men who had been asked to serve on these Committees by themselves. If the amendment were passed, within three or six months every man who was on these Committees would be quite prepared to come in under any resolution the Conference might like to pass. As to whether men serving on these Committees rendered any service to non-panel men, he thought that their service on these Committees was absolutely futile, but a little time should be given to ask them to come off.

Dr. Oppenheimer, Hampstead, thought it was wise on the present occasion to make the limit as wide as possible.

Dr. Southcomb, City of London, stated that a large number of members in London had signed Form 43 under the Insurance Act. If the resolution excluded these, they would have to retire, though it would be more in sorrow than in anger. The vast majority of the non-panel men in London had signed certain of these agreements under the Act. (Cries of dissent.) He could only repeat that nearly all the non-panel men in East and North-East London had signed these forms; the resolution would exclude all these from membership.

Dr. Pinder, Manchester, remarked, amid laughter, that he had never seen or even heard of the Form that allowed contracting out.

Dr. Trotter, Huddersfield, thought the main question was whether a man on an Insurance Committee could do any good; presumably such a man went on the Committee with the idea of assisting the working of the Act, otherwise he had no right to go on the Committee at all. Mr. Turner had given only one complete instance in which good had been done by such service. The mover and the seconder of the amendment had to a large extent taken up entirely opposite views. They had heard from Dr. Montgomery Smith the statement that after six months these men would be tumbling head over heels to come off, but they had from Mr. Turner the statement that it was desirable that they should stay on these Committees until the rearrangement took place, a couple of years hence. His experience was that service on these Insurance Committees had a disastrous effect on the medical men who adopted it. He had been in the thick of this fight from the beginning, and the one thing that had been their undoing all through was compromise—compromise was the curse of the British Medical Association. If this organisation which they were now going to launch was to have anything to do with the British Medical Association, he wanted nothing whatever to do with it. They might lose a few men by taking a strong attitude, but the men they would lose would be men who were prepared to temporise.

Dr. Lowe, Crewe, confessed that he heard Mr. Turner's speech with disappointment, remembering a speech he made a year ago. On the former occasion he heard Mr. Turner quote *Timeo Danaos et dona ferentes*, and he had heard him say that they should have absolutely nothing to do with anybody who had anything to do with the Insurance Act. He appealed to the meeting not to desert high principles for the sake of detail. He could not see that it was logical in a state of warfare for a man to be in the councils of the enemy.

Dr. Beaton expressed the opinion that Insurance Committees were not doing much for anybody except the panel doctors. There were supposed to be a million and a half insured persons in London, and of these only about 1,500 had been allowed to contract out, and were being attended by doctors who had signed Form 43; that was the one thing that was being done by the Medical Members of the London Insurance Committee, and it was hardly worth doing.

Dr. MacHeath, Southampton, raised the point of a non-panel doctor attending a member of such an organisation as the Seamen and Firemen's Union, which came under the Insurance Act. The resolution would prevent such a man from joining the Federation.

Dr. H. B. Greene said that Form 43 was merely brought out to break down the London resistance; the doctors who signed it had to accept the same conditions as panel doctors, except with regard to payments. He would give two instances to show how useless the presence of doctors was on Insurance Committees. One was that the Worcestershire County Committee endorsed a resolution allowing herbalists to act under the Insurance Act. The other instance was what occurred at Wisbech, where five medical men were persecuted and their houses stoned by an organised political mob, acting practically with the concurrence of the Insurance Committee of the district.

Dr. Macnamara, Non-panel Medical League, said that if the organisation were to be a success it must be on the basis of meeting the requirements and wishes of the majority. He was prepared to accept that position, but he asked that if the decision was in favour of Mr. Turner's amendment that decision also should be accepted as final. Personally he would favour leaving the organisation open to all non-panel men, whether on Insurance Committees or not, provided they worked wholeheartedly with the organisation.

Dr. Wheeler Hart strongly disapproved of the amendment, and said he had not heard a single convincing instance of a Medical Member of an Insurance Committee having influenced that Committee. The Medical Members of these Committees had no power whatever, and non-panel men joining these Committees were doing a great disservice by expending their energies in trying to push down a wall which they could not push down. Moreover, the presence of their names on these Committees lent countenance to the Act in the eyes of the public. He attached no importance to the argument raised about doctors who treated contracted-out patients. Lord Justice Eady, in a recent decision, laid it down that this was practically the same as private insurance.

Dr. Biggs, Wandsworth, objected to excluding from the Union members of the profession who had merely done what they were asked to do by certain of their professional brethren.

Dr. Johnston, Brighton, maintained that doctors on Medical Committees could do good work for the profession. At the same time, if the Conference did not pass the amendment he would at once send in his resignation as a member of the East Sussex Medical Committee.

Dr. O'Sullivan, Liverpool, said they had come together in order to federate, and they were being watched by the British Medical Association and all over the country; surely it was not impossible for them to leave that room united? Merely because some of them were on these Committees were they going to endanger the organisation? By "non-panel" was meant no service under the Act. Let them stick to their guns and they would come out right.

### THE VOTING AND RESULT.

The closure having been moved and carried,

Mr. Turner's amendment was put to the meeting, the voting being by card, according to the membership strength of Associations. After a careful scrutiny and recount,

The Chairman announced the result of the voting as follows:—

| | |
|---|---|
| Against the amendment ... ... | 530 |
| For the amendment ... ... | 525 |
| Majority against the amendment | 5 |

The original resolution was therefore before the Conference.

Dr. Evan Jones proposed a further amendment: "That the principal condition of membership of the new Federation shall be that applicants should not be on the panel directly or indirectly, and that all further conditions be left to the decision of the Associations forming this Federation."

Dr. Carswell protested that the amendment was narrower even than Mr. Turner's amendment, which the Conference had rejected.

Dr. Porter said he could not vote for any amendment; he had no authority to do so, and he might say that he represented practically all the non-panel men of Scotland.

After some further desultory discussion a short conference took place between the leaders of diverse views, and it was eventually agreed that the amendment should read as follows:—

**"That the principal condition of membership of the new Federation shall be that applicants for membership shall not be on the panel, directly or indirectly, and that all ocal Associations shall have power of autonomy."**

The amendment was carried with two dissentients, and was then put and agreed to as a substantive resolution.

Dr. Porter said he wished it to be understood that this was, of course, provisional as far as Scotland was concerned. He had to take the opinion of his constituents.

### THE TITLE OF THE FEDERATION.

On the motion of Dr. J. Brassey Brierley, seconded by Dr. H. B. Woodcock, it was agreed:

**"That the name of the Federated Societies shall be the National Medical Union."**

### SUBSCRIPTION.

On the motion of Dr. F. Porter, seconded by Dr. O'Sullivan, Liverpool, it was agreed that the subscription should be one guinea annually per member.

### RELATIONS WITH BRITISH MEDICAL ASSOCIATION.

Dr. Edwin Smith proposed and Dr. G. B. Wilson seconded:—

"That while membership of the British Medical Association shall not disqualify for ordinary membership of the Federation, no official of the Federation shall be at the same time on any central executive committee of the British Medical Association."

Mr. E. B. Turner, while opposing the resolution, sympathised with the motives of those supporting it, and he said further that members of the Council of the British Medical Association did not intend to stand for the executive of the new body, nor would they accept service upon it if they were asked to do so.

The feeling of the meeting being clearly in sympathy with the resolution, the Chairman suggested that, now that the matter had been ventilated, it should not be further discussed.

Dr. Edwin Smith, acting on this suggestion, and having heard the remarks of Mr. Turner, withdrew the resolution.

### HONORARY OFFICERS.

The Conference unanimously elected the following provisional officers and Council:—

*Presidents.*

Mr. G. A. Wright, F.R.C.S., and Professor Russell (Edinburgh University).

***Vice-Presidents.***

Dr. John Playfair (President of the Edinburgh Medical Guild), Mr. William Coates, C.B., D.L. (Chairman of the Conference), Dr. Bernard O'Connor (Barrister-at-Law and Member of the London Medical Committee), and Dr. Greenyer (Brighton).

*Treasurers.*

Dr. Brassey Brierley (Manchester) and Dr. H. Oppenheimer (Hampstead).

*Secretaries.*

Dr. R. Carswell (Wandsworth) and Mr. E. Arthur Dorrell (Secretary of the London Medical Committee).

### PROVISIONAL FEDERATING COUNCIL.

Drs. C. E. M. Lowe and H. B. Greene, National Medical Union; Drs. McNamara and S. Lee, London Medical Committee; Drs. Burgess and G. D. Wilson, Wandsworth; Drs. J. Ford Anderson and H. Sharman, Hampstead; Drs. Shoyer and Bousfield, Islington; Dr. Hemmans, Lewisham; Drs. Spurgin and Macevoy, Non-Panel Medical League, Willesden; Drs. Porters, T. J. Thyne, Torrance Thompson, and James Smith, Edinburgh; Dr. Damer Harrison, Liverpool; and Drs. T. Johnson and Baskin, Brighton and East Sussex.

### OFFICES (pro tem.).

The Waldorf Hotel, Strand, London, W.C.

### VOTES OF THANKS AND SYMPATHY.

On the motion of Dr. O'Sullivan, Liverpool, seconded by Mr. E. B. Turner, the Conference unanimously voted its sympathy with Dr. Meacock and the other practitioners at Wisbech who had been subjected to suffering and annoyance in connection with the working of the Insurance Act.

The Conference also decided to convey its sympathy to German confrères in their struggle against the German Insurance System, and Dr. Oppenheimer was requested to deal with the matter.

On the motion of Dr. Greene, seconded by Dr. Oppenheimer, thanks were voted to Sir Henry Burdett for the manner in which he had opened the columns of The Hospital to aid non-panel interests.

Sir Henry Burdett moved that the best thanks of the Conference be given to Mr. Coates for his conduct in the chair. He congratulated the Conference on the success which had attended it; he believed that if they stood together and went forward they would lead the medical profession, and no other body of the profession could lead without them. The success of the Conference had been due to their Chairman more than to anyone else, and he proposed that their thanks should be formally engrossed and presented to him.

Dr. Johnston (Brighton) seconded the motion, which was adopted with much cordiality.

The Chairman returned thanks.

On the motion of Dr. Hart, seconded by Dr. Clarke (Manchester), the thanks of the Conference were also voted to Dr. Prowse and Dr. Carswell for their work in arranging the preliminaries of the Conference.

The Conference then terminated.

